# Atlanta Medical and Surgical Union

**Published:** 1884-10

**Authors:** 


### ATLANTA MEDICAL AND SURGICAL UNION.

REPORTED FOR THE ATLANTA MEDICAL AND SURGICAL JOURNAL.

STATED MEETING, September 2d, 1884.

Dr. W. S. Elkin, President, in the chair.

The regular paper for discussion, which was to have been read by Dr. J. C. Avary, was postponed in consequence of Dr. Avary's absence.

The President called for a report of cases and asked Dr. J. D. Wilson if he had a case to report. The Doctor then reported the following case:

In the latter part of May last I was called at midnight to see a negro patient said to be dying. Arriving at the bedside, I found a man, nearly sixty years old, presenting all the characteristic symptoms of Hemiplegia. His face was drawn noticeably to the right side, the tongue, on protrusion, pointed to the left, and the left arm and leg were completely paralyzed. About two o'clock in the afternoon of the same day, without apparent premonitory symptoms, he had fallen to the floor in one of the city drug stores and had been taken home in a hack. His answers to my questions betrayed a fair intelligence and a tolerably correct understanding of his situation. At no time had consciousness been abolished, and yet his mind seemed to act somewhat spasmodically, if I may be allowed the term. He was able to think accurately when aroused, but when drifting along without effort was undoubtedly flighty. His pulse was small and thready in the extreme, breathing labored, and the abdomen as tight as a drum from gaseous distension. I hardly expected him to live till morning, but prescribed digitalis and aromatic spirits of ammonia, and ordered small quantities of whisky at short intervals. The next day his condition was practically the same, and for three or four days death seemed so imminent that the above stimulating treatment was continued with the mere idea of keeping him alive. In a short time, however, an improvement was noticed, and as he gradually gained in strength the treatment was changed to iodide of potassium in constantly increasing doses. The history of some venereal lesion, dating back ten years, was made out, but his recollection was so vague and his answers so variable that I could never satisfy myself of its undoubted specific character. If it was a primary syphilitic lesion his subsequent symptoms had certainly been slight. His system was quite intolerant of the iodide and he was never able to take as high as twenty grains three times daily without the induction of such painful symptoms that I was obliged to reduce the dose. The bichloride of mercury was also exhibited for a time in conjunction with the above. In about six weeks he was able to move slightly the toes of his left foot, and was soon able to lift the left leg over the right as he sat in his chair. The muscles of the face and tongue regained their power, but he was never able to move the affected arm in the slightest degree. The left arm and hand were also markedly œdematous from the first. The tympanitic condition of the bowels persisted throughout. His appetite was fair and he had regularly two stools daily during my term of treatment. He had been a regular drinker through life, and was allowed a considerable quantity daily. Drs. Gray and Gaston saw the case with me at different times; the former recommending strychnia as a nerve tonic and the application of a blister to the spine, the latter an increase in the dose of strychnia. These suggestions were carried out and the patient got along moderately well until about the middle of August, when a general anasarca set in which puffed him up like a football from head to foot, and after lingering for a few days in this condition he died. Drs. Gaston, Greer and myself examined the brain after death and found no tumors, syphilitic or otherwise, within the cranium. The brain substance was of good consistence until we came to the corpora striata. That on the left side was hard, firm and normal; that on the right side was discolored a dark brown and had the appearance and consistence of the substance of a rotten apple. Thus our examination renders it highly probable that an embolus, passing with ease up the common carotid, was firmly caught and permanently arrested by the smaller calibre of the middle cerebral artery.

Dr. J. G. Hopkins reported an interesting case of stricture of the urethra, which had caused such serious nervous trouble that he had gone North with the patient and consulted a prominent neurologist of New York city, who had diagnosed the case as one of congestion of the brain, and who assured him that it could be cured in a few days. After four or five days' treatment, in which the patient was given the same remedies he had been taking before leaving home, he was pronounced well by the New York specialist, when in reality the patient was in a worse condition than when he began treatment. The doctor's remarks produced a great deal of laughter as he minutely described the celebrated specialist and his method of treatment.

Dr. W. F. Westmoreland gave his experience with the same specialist, and stated that a patient of his had accidentally fallen into this same specialist's hands and had been treated for "serious brain trouble," as he called it, when Dr. Sayre, of New York, saw the case and found that the trouble was in the sacro iliac joint, which he treated and the patient was soon relieved.

Dr. Westmoreland also reported a case of trachæotomy, for the removal of a foreign body that had remained in the trachae for thirty-six hours. The Doctor stated that the larynx was so much inflamed that he did not close the wound in the trachae for twenty-four hours. The patient made a good recovery.

Dr. W. B. Parks reported a case of hemorrhage from the mouth and umbilicus in a child three weeks old. The hemorrhage continued for three days. No application seemed to have any effect towards arresting it. The child died on the third day.

The president stated that Dr. Jno. Thad. Johnson, a member of the Union, would soon move to California, and that he thought the Union should take some action on the matter.

On motion, a committee consisting of Drs. W. S. Elkin, R. O. Cotter and J. G. Hopkins were appointed to consider the matter.

---

STATED MEETING, Sept. 16th, 1884.

Dr. W. S. Elkin, President, in the chair.

Dr. Geo. H. Noble reported the following case:

### UNUSUAL SUCCESS IN AN UNFAVORABLE CASE OF OVARIOTOMY—NEW ANTISEPTIC DRESSING.

The interest for which this operation is reported is the unusual good recovery in such an unfavorable case, and the antiseptic and absorbent cotton with which the wound was dresssd.

The tumor was of seven years' duration, weighed twenty pounds, and held four gallons of fluid.

With the assistance of Drs. V. H. Taliaferro, R. O. Cotter, J. D. Wilson and W. W. Greer I removed it through a four and a half inch incision, tied pedicle with Tait's knot, returned it to the abdominal cavity and closed the wound completely. No drainage.

The tumor was very strongly bound down in the pelvis by numerous bands that required great force to tear up, and quite extensively to the abdominal walls, especially where it had been tapped three years before, to the fundus of the bladder and to the great omentum by less firm union. Many strong bands passed from side to side of the pelvic cavity which had of necessity to be broken off before thorough douching and sponging could be effected. This gave rise to considerable oozing which required time to suppress. The pedicle at the base of the tumor was a little triangular and a little more than three inches wide, very vascular, and engorged, the greater part of the fallopian tube was removed with the tumor.

At no time did the temperature go above 100 deg. or pulse above 96 per minute from the effect of the operation.

The wound was dressed simply with dry absorbent and antiseptic cotton (that I make myself) and left untouched until I removed the sutures on the sixth and seventh days, when the wound was completely adhered throughout and not a drop of pus or moisture was to be found—even the sutures were free.

I used the non-antiseptic ligature and suture. The only antiseptic used was a little carbolic acid in the first reservoir of water that the cavity was douched with. The second douche contained none. The after treatment consisted of calomel and citrate of magnesia to move the bowels, and morphine to secure quiet and rest. Considering all things she made an unusually good recovery, for she had practically passed the effect of the operation in a week's time though not deemed sufficient for removal until the third week.

### ABSORBENT AND ANTISEPTIC COTTON.

This, though simple, is effectual. We have tried it in cases at our private infirmary, and Dr. Taliaferro and myself use it daily. Of its tests or trials I hope that a reference to the foregoing ovariotomy in a woman of a tubercular diathesis, and one other operation, the removal of a malignant breast from a very emaciated and debilitated woman, who for a number of years had been an invalid and had a constant tendency to suppurative discharges and distressing eczema from all abrasions of the skin, will be sufficient. We left the suture intact twenty-three days, also a ligature in the centre of the wound, the incision extending far enough to remove the axillary gland, and not a drop of pus was to be found.

Though the germ theory has been established, and the process of putrefaction is dependent upon the admission of some form of the many living organisms, we do not coincide in the belief that the introduction of these germs will set up putrefaction in every instance but only in those cases whose vital powers are in such a low state as to break down and furnish dead material for them to live upon. Yet that we may avail ourselves of what good there is in antiseptics we have prepared this cotton and devised this mode of dressing.

The cotton is first boiled for a number of hours in a strong alkali: Bi-carb. soda, 4 zo to the gal., then thoroughly rinsed and reboiled in clear water; this is followed by boiling in a very strong solution of permanganate of potassium, 1 oz. per gal.; compress, dry and card into bats for use.

We have heretofore dressed the wound by simply placing the cotton over it and applying a cloth bandage, but shall hereafter glue the edges of the cotton bats to the skin to prevent the possibility of any germs entering between it and the cotton. For this purpose we judge the flexible collodion to be all we desire.

This dressing, dusted with iodoform (scented with oil of citronella), makes the simplest and most perfect antiseptic dressing that I have seen. It surpasses the Lister dressing, for I have never yet observed any blood or secretions to decompose where this cotton was used, no matter how well saturated it be.

It will be observed from the mode of application that any germs which come in contact with the wound must of necessity pass through the antiseptic medium, the action of which renders them more or less inert. We therefore exclude all deleterious elements of the atmosphere and get here an exit through the cotton for all bleeding or secretion that may take place.

In the ovariotomy above mentioned there was bleeding of the wound from vomiting, which the cotton absorbed even from the wound itself with the result as above stated.

We do not claim it to be a perfect dressing, or that it invariably insures success, but that it is the best antiseptic dressing that I have seen tried, (Lister's and others) and especially as in connection with the iodoform.

Dr. Greer reported a case of a scalp wound where the hemorrhage was so profuse that measures had to be resorted to to arrest it. It was impossible to secure the artery from which the blood flowed, and he resorted to packing the wound with cotton saturated with tincture of iron. This arrested the hemorrhage at once. This was allowed to remain for several days, when it was removed, and quite a quantity of pus found in the wound. The pus had burrowed for a considerable distance in every direction. Under the use of a carbolized wash the wound soon regained a healthy appearance and was now getting well.

Dr. C. C. Quillian reported a case of transverse fracture of patella-

Was called to see Mr. B., aged 46, Irishman. Found him suffering considerable pain in right knee with complete loss of power of leg. Examined the knee and found patella fractured a little below the middle, with the fragments separated an inch or more. Placed the limb on a long posterior splint, with foot piece well padded.

Adjusted the fragments and bound the limb to the splint with a roller bandage, making a figure of 8 turn around the knee to hold the fragments in position.

Directed that the joint be kept cool with ice water and left it in that position for 3 or 4 days. Then removed the bandage and splint, and without ever allowing the limb to be flexed applied a plaster of Paris bandage extending from the toe to the hip joint making a few turns around the pelvis to hold the bandage in position. This bandage remained for three weeks, when it began to break and was removed and another of the same material applied—being careful not to flex the limb in its application.

The last bandage remained till the twelfth week from the time of injury, when it was removed and *bony union* found to have taken place. The patella was somewhat enlarged, being a little longer from above downwards than the other; but movable from side to side over the knee joint. The knee joint was at first very stiff, allowing of only slight flexion; but by manipulation it has regained its mobility and now can be flexed till the heel almost touches the nates—making a very serviceable limb with a strong knee joint. The patient experiences no discomfort whatever from the injury, the limb performing all its functions with ease.

I consider the result of bony union due to the fact that the limb was never flexed during the entire treatment.

From the application of the first plaster of Paris bandage, the patient was allowed the use of crutches and walked about the house and yard daily, with the foot supported by a sling passed around the neck and one shoulder.

The committee appointed to draft resolutions concerning the tendered resignation of Dr. J. T. Johnson and his proposed departure for California, submitted the following:

WHEREAS, The resignation of Dr. John Thad Johnson has been received, and his intention of leaving the city made known, be it

*Resolved*, That the resignation be accepted with great reluctance, knowing, as we do, that we thus sever a connection with an efficient member, whose ability as a surgeon, medical writer and lecturer is marked and widely recognized.

His ardent devotion and patriotic labor for the Georgia State Medical Association won for him at an early age the highest office it had power to bestow, and his years of teaching in our medical colleges stamp him as a man of indomitable energy and boldly attest his superior qualifications.

Medically and socially he is the peer of any man, and is loved most by those who know him best. We regret to give him up, and in so doing realize that Georgia's loss will be the gain of his adopted State. We cheerfully commend him to the good people of the Pacific slope as one entirely worthy of their medical trust, and bespeak for him that full share of patronage which he so eminently deserves.

W. S. ELKINS, M. D.,<br>

J. G. HOPKINS, M. D., *Committee.*<br>

R. O. COTTER, M. D.,

